# The influence of citrus aurantium and caffeine complex versus placebo on the cardiac autonomic response: a double blind crossover design

**DOI:** 10.1186/s12970-018-0240-0

**Published:** 2018-07-24

**Authors:** Brian Kliszczewicz, Emily Bechke, Cassie Williamson, Paul Bailey, Wade Hoffstetter, John McLester, Cherilyn McLester

**Affiliations:** Department of Exercise Science and Sport Management, Kennesaw State University, Kennesaw, GA USA

**Keywords:** P-synephrine, Heart rate variability, Caffeine, Anaerobic, Recovery

## Abstract

**Background:**

The purpose of this study was to examine the resting cardiac autonomic nervous system’s response to the ingestion of a complex containing *Citrus aurantium* + Caffeine (CA + C) and its influence on recovery following a high-intensity anaerobic exercise bout in habitual caffeine users.

**Methods:**

Ten physically active males (25.1 ± 3.9 years; weight 78.71 ± 9.53 kg; height 177.2 ± 4.6 cm; body fat 15.5 ± 3.13%) participated in this study, which consisted of two exhaustive exercise protocols in a randomized crossover design. On each visit the participants consumed either a CA + C (100 mg of CA and 100 mg of C) or placebo (dextrose) capsule. After consumption, participants were monitored throughout a 45-min ingestion period, then completed a repeated Wingate protocol, and were then monitored throughout a 45-min recovery period. Cardiac autonomic function (Heart Rate (HR) and Heart Rate Variability (HRV)) and plasma epinephrine (E) and norepinephrine (NE) were taken at four different time points; Ingestion period: baseline (I1), post-ingestion period (I2); Recovery period: immediately post-exercise (R1), post-recovery period (R2). Heart rate variability was assessed in 5-min increments.

**Results:**

A repeated measures ANOVA revealed significant time-dependent increases in HR, sympathetic related markers of HRV, and plasma E and NE at I2 only in the CA + C trial (*p* < 0.05); however, no meaningful changes in parasympathetic markers of HRV were observed. Participants recovered in a similar time-dependent manner in all markers of HRV and catecholamines following the PLA and CA + C trials.

**Conclusion:**

The consumption of CA + C results in an increase of sympathetic activity during resting conditions without influencing parasympathetic activity. CA + C provides no influence over cardiac autonomic recovery.

## Background

The cultivation of commercially available supplements has substantially increased throughout recent years, making the use of pharmacologic ergogenic aids more prevalent and readily available to the general population and athletic community. In general, ergogenic aids purport to contain individually unique properties that result in various physiological outcomes (e.g., metabolic); however, a majority of these claims are overstated or not fully understood. Recently, a growing interest in the combined supplementation of *Citrus aurantium* (CA) and Caffeine (C) has emerged due to its ability to decrease rate of fatigue during exercise and increase metabolic rate [1, 2].

While examining these supplements individually, C has been known to stimulate the central nervous system (CNS) (i.e. increase heart rate, cognitive function, blood pressure) which in turn increases motor function, availability of plasma free fatty acids, and increased performance outcomes during endurance exercise [3–5]. Whereas CA is most popularly used as a weight loss supplement, which is attributed to its primary protoalkaloidal constituent, p-synephrine [6–8]. P-synephrine has an affinity to ß-3 receptors and therefore enhances lipolysis and thermogenesis. CA was previously theorized to produce similar effects on the CNS as ephedra due to p-synephrine’s similarities in chemical structure. However, it has recently been found that the influence of CA over CNS appears to be minimal, although not fully understood [6]. To this point, the literature is conflicting in regards to the magnitude of the effect of CA on the cardiovascular system, especially when combined with C [7, 9, 10].

A study conducted by Ratamess et al., [2] supports the notion that when combined, caffeine (100 mg) and p-synephrine (100 mg) can increase local muscle endurance within multiple sets during resistance training. The findings of this study are suggestive of enhanced recovery between multiple sets [2], which may therefore translate to the recovery of other mechanisms of physiological stress and control. The analysis of the autonomic nervous system (ANS) is a viable method to assess transient alterations and stress on the body, and may also provide insight into systemic readiness [11]. The homeostatic condition of the body is maintained through the sensitivity and the responsiveness of the ANS to internal and external stimuli [11, 12]. Fluctuations of the ANS are observed through changes in its two branches; the sympathetic nervous system (SNS) and parasympathetic nervous system (PNS). ANS activity can be indirectly measured through the observation of cardiac autonomic activity (e.g., vagal and sympathetic modulations), which can be measured through heart rate variability (HRV). In conjunction with HRV measures, plasma catecholamines, epinephrine (E) and norepinephrine (NE), provide direct markers of SNS activity and allow for a more holistic view of ANS function.

To the authors’ knowledge, no known research has been conducted examining the influence of CA + C complex supplementation on cardiac autonomic activity and recovery following a high-intensity exhaustive exercise protocol. Improvements in acute ANS recovery may translate to reduce transient stresses within the CV system as well as prevent systemic over reaching [11, 13, 14]. Therefore, the purpose of this study was to examine the resting ANS response to the ingestion of CA + C as well as its influence on recovery following an exhaustive anaerobic exercise protocol.

## Methods

### Participants

Fourteen apparently healthy males who habitually consume caffeine (95–300 mg serving per day, at least 4 days a week) were recruited for this study. Prior to participation, all individuals were made aware of the procedures and risks associated with the study and signed an informed consent. A health history questionnaire (HHQ) and physical activity readiness questionnaire (PAR-Q) were administered in order to ensure that participants were capable of engaging in vigorous physical activity without physician’s clearance as defined by the guidelines provided by the American College of Sports Medicine [15]. Any individual who reported having orthopedic conditions, cardiovascular, pulmonary, or metabolic disease were excluded from the study. Those who regularly over consumed caffeine (≥ 300 mg/day) were also excluded from the study.

Physical activity inclusion criteria required all participants to engage in at least three-days of aerobic training and two-days of resistance training per week for the previous six months. Participants were recruited via word of mouth from the local metropolitan area. Prior to all sessions, participants were asked to wear light and comfortable clothing, fast for a minimum of four-hours, avoid exercise for 24 h, and avoid caffeine consumption for 12 h. The Institutional Review Board approved all testing procedures and protocols prior to beginning data collection.

### Experimental design

The study was performed in a double-blind, placebo-controlled, randomized crossover fashion in which only one investigator knew the contents of the supplementation; this investigator was not involved in the collection or analysis of the study outcome measures. Participants were asked to attend two separate sessions in the exercise physiology laboratory, with both visits occurring within a nine-day period and a minimum of 72-h between visits. All visits were performed between 5:00 am 7:00 am. The first visit consisted of obtaining informed consent, PAR-Q, HHQ, and anthropometric measures. Height (cm) and weight (kg) were collected using an electronic physicians scale (Tanita WB 3000, Arlington Heights, IL) and body fat percent (BF%) was collected via a dual-energy x-ray absorptiometry scan (GE Lunar iDXA, Madison, WI).

**Fig. 1:**
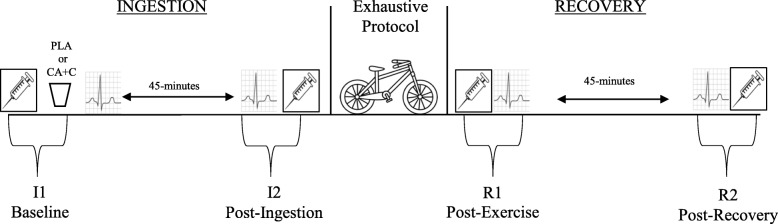
Study Design

The remaining two visits can be described in two overarching sections; the ingestion period with pre and post time points (I1 & I2) and the recovery period with pre and post time points (R1 & R2). The ingestion period consisted of baseline measures (I1), which included veinipuncture and the fitting of a polar heart rate monitor, followed by the consumption of either the supplement (CA + C) or placebo (PLA). The 45-min ingestion period was initiated after the participants consumed CA + C or PLA. Upon the completion of the ingestion period, a post-ingestion venipuncture were performed (I2). Participants then performed a standardized warm-up prior to initiating the anaerobic exhaustive exercise protocol. Immediately following the exercise protocol a post exercise venipuncture was performed (R1). Then, participants were monitored throughout a 45-min recovery period. At the end of this recovery period the final venipuncture was taken (R2). Cardiac activity was continuously recorded during the 45-min ingestion and recovery periods. Analysis of these recordings were made in 5-min segments beginning at the 5-10th minutes of the ingestion and recovery periods. These values are present as time points I1 & R1 (respectively). Additionally, the 40-45th minutes of the ingestion and recovery periods and are present as time points I2 & R2 (respectively). The study design can be seen in Fig. 1.

### Exhaustive exercise protocol

Upon the completion of the 45-min ingestion period, participants were allotted a seven-and-a-half minute warm up on a Monark ergometer (Monark 828E Ergomedic Test Cycle, Vansbro, Sweden) while pedaling between 50 and 100 rpm at a resistance of 1.5 kp. Participants were immediately walked to an electronically braked cycle ergometer (Sport Excalibur, Lode BV, Groningen, The Netherlands), where the bike was adjusted to the appropriate settings in order to ensure the knee was at a slight bend at the bottom of the revolution. Bike settings were repeated for both trials. Following the appropriate adjustments, participants feet were strapped into the pedals and the protocol was initiated. The start of the exhaustive exercise protocol comprised of a one-minute warm-up period performed at 50 W with a rolling start into the Wingate test. Each Wingate test was 30-s in duration and participants were encouraged to pedal at their maximal effort against a resistance of 0.80 Nm/kg [16]. There was a total of three Wingate tests performed with a two-minute active recovery period between each test. The active recovery was a self-selected pedal rate against a resistance of 50 W and a rolling start into the subsequent Wingate test. At the completion of the last Wingate test, participants were walked to a separate room to undergo a post exercise venipuncture and to begin the measurements of cardiac autonomic recovery measures (R1-R2). Pre-testing protocols on the electronically braked cycle ergometer followed manufacturer guidelines.

### Blood collection and analysis

A trained phlebotomist drew six milliliters (ml) of blood via the antecubital vein during four-time periods throughout the study: I1, I2, R1, R2 (Fig. 1). Blood draws were collected in lithium heparin tubes and inverted based on the manufactures’ recommendations prior to centrifugation. Samples were centrifuged at 2500 rpm for 15-min, then aliquoted and stored in a − 80 °C freezer until subsequent assay analysis. Plasma samples were assayed for E and NE using commercially available ELISA kits (Abnova, Taoyuan City, Taiwan). In order to account for the plasma volume shifts following the exercise bout, all samples were normalized by using the established protocols of Dill and Costill [17]. Hematocrit (Hct) and hemoglobin (Hb) were collected via finger sticks at each venipuncture time point (Alere Hemopoint 2).

### Heart rate variability collection and analysis

Heart Rate Variability is a non-invasive measurement that quantifies the timing between consecutive R-R intervals. The measurements are derived from an electrocardiogram or HR detection device (i.e. HR monitors) [18]. Heart Rate Variability and HR recordings were collected using the Polar® monitor system and transferred to the Polar Team^2^ software (Lake Success, NY). Heart rate monitors were positioned under the sternum against bare skin. Throughout each 45-min recording period, participants were seated in a quiet, dimly lit room with no external stimuli.

Analysis was completed through the online Kubios Software (Kubios V 2.2, Joensuu, Finland) wherein recordings were transformed into a tachogram, which plots the successive R-R intervals (y-axis) against the number of beats within the time series (x-axis). Heart Rate Variability markers were analyzed in five-minute segments during the beginning (5–10 min: I1, R1) and end (40–45 min: I2, R2) of the ingestion and the recovery periods. During analysis, an artifact correction of “low” with a sensitivity set to identify any R-R abnormality ±0.35 s was applied using a piecewise cubic spline interpolation method in order to filter additional artifact noise present in the tachogram (Kubios V 2.2, Joensuu, Findland) [19, 20]. Any segments that contained three or more irregular R-R intervals were excluded from analysis.

The markers chosen for this study were the time domain indexes of the root mean square of successive differences (RMSSD) and the standard deviation of normal-to-normal intervals (SDNN); the frequency domain measures of High Frequency Power (HF) (0.15–0.40 Hz), normalized High Frequency Power (HFnu), Low Frequency Power (LF) (0.04–0.15 Hz), normalized Low Frequency Power (LFnu), and their ratio LF/HF. The Fast Fourier Transformation was applied to the frequency domain makers. Frequency domain measures come with inherent limitations related to ANS interpretation due to sensitivities to breathing frequencies and therefore will be assessed along with time domain measures [21]. RMSSD and HF are widely recognized as markers of vagal activity [18, 22], while SDNN and LFnu are believed to provide insight into SNS influence, though they possess activity from the PNS [23, 24]. LF/HF ratio provides insight into ANS balance [25]. All R-R interval recordings were measured using a window width of 256 s and overlap of 50% through the specialized HRV software (Kubios V 2.2, Joensuu, Findland).

### Supplement preparation

CA and C powder were purchased from Blackburn distributions (Caffeine powder, Blackburn distributions limited, Nelson Lancashire, England; *Citrus aurantium* powder, Blackburn distributions limited, Nelson Lancashire, England). The PLA contained 200 mg of dextrose, whereas the supplement contained a combination of CA (100 mg) and C (100 mg). Each component was measured using an electronic supplement scale and encapsulated in green, non-translucent, size zero gelatin capsules. The identity of the content within the capsules was not revealed until all data were collected and statistical analyses were completed.

### Statistical analysis

All data were analyzed using the statistical software package SPSS (SPSS, Version 24 for Mac, Chicago, IL). A Shapiro-Wilk test was performed to examine the normality of distribution on the HRV markers: RMSSD and SDNN. In order to assess changes in time within trial (CA + C/PLA) repeated measures analysis of variance (ANOVA) were run (Ingestion: I1 vs. I2; Recovery: R1 vs. R2) in cardiac autonomic markers (HRV and HR) and plasma catecholamines (E and NE). Significance for all statistical analyses was set at ≤0.05. The data is presented as the mean ± standard deviation (SD). In order to determine the effect size, the recommended guidelines of Quintana were used. [26]. Thresholds for effect size were the following; a small (< 0.25) moderate (0.50), and large effect (0.90).

## Results

Four participants were removed from the study due to adverse reactions to the phlebotomy procedure (i.e. vaso-vagal reactions) (*n* = 2), pain brought on by brochiospasm (*n* = 1), and the presence of more than three irregular R-R intervals in the recordings (*n* = 1). Therefore, a total of ten physically active males completed the study. Participant characteristics can be seen in Table 1. Additionally, normality was violated in several HRV markers and therefore the natural logarithmic transformation (ln) was applied prior to further statistical analysis: RMSSD (lnRMSSD), SDNN (lnSDNN), HF (lnHF), LF(lnLF).

**Table 1:** Participant Characteristics (*N* = 10)

Characteristic	Mean ± SD
Age (y)	25.1 ± 3.8
Height (cm)	177.2 ± 4.6
Weight (kg)	78.8 ± 9.4
Body Fat (%)	15.5 ± 3.0
Caffeine/day (mg)	209 ± 95.5

During the PLA ingestion phase, no significant (*p* > 0.05) time effects were found in any cardiac autonomic markers or catecholamines except for a significant decrease in HFnu and a significant increase in LFnu (*p* < 0.05). However, during the CA + C trial ingestion period, a significant time effect (p < 0.05) was observed from I1 to I2 with increases in HR, lnSDNN, LFnu, NE, E, and a decrease in HFnu. While examining the recovery period in the PLA and CA + C trials, no significant time effects occurred in HFnu, LFnu, or LF/HF. A significant decrease in HR, lnRMSSD, and lnSDNN occurred along with a significant decrease in E and NE. Means ± SD and effect size (Cohen’s d) can be seen for the ingestion period in Table 2A and the recovery period in Table 2B.

**Table 2:** Markers of ANS activity during the Ingestion (A) and Recovery Periods (B)

A. Ingestion Period	PLA				CA + C			
	I1	I2	*p*-value	Cohen’s d	I1	I2	*p*-value	Cohen’s d
Heart Rate (bpm)	62.6 ± 14.34	65.5 ± 10.96	0.60	0.23	59.7 ± 7.93	64.6 ± 9.13	0.04	0.57
lnRMSSD (ms)	3.95 ± 0.49	3.87 ± 0.40	0.57	0.17	4.0 ± 0.50	4.08 ± 0.45	0.45	0.16
lnSDNN (ms)	4.32 ± 4.40	4.40 ± 0.33	0.59	0.22	4.36 ± 0.48	5.04 ± 0.56	0.03	1.28
lnHF (ms^2^)	6.65 ± 1.00	6.55 ± 0.85	0.70	0.12	6.95 ± 0.79	7.04 ± 0.82	0.70	0.11
HFnu	42.92 ± 16.87	29.37 ± 16.06	0.01	0.82	39.01 ± 17.13	31.78 ± 12.49	0.03	0.48
lnLF (ms^2^)	7.00 ± 1.00	7.51 ± 0.73	0.21	0.58	7.43 ± 1.05	7.86 ± 0.71	0.05	0.48
LFnu	57.01 ± 16.89	70.62 ± 16.07	0.01	0.82	58.95 ± 20.45	68.20 ± 12.50	0.03	0.55
LF/HF	2.08 ± 2.29	3.20 ± 1.54	0.07	0.57	2.40 ± 2.31	2.75 ± 1.63	0.45	0.18
E (nmol/L)	4.40 ± 2.93	3.97 ± 2.05	0.41	0.17	3.53 ± 2.08	4.96 ± 2.84	0.02	0.58
NE (nmol/L)	21.56 ± 5.57	23.40 ± 9.63	0.51	0.23	18.16 ± 3.91	28.29 ± 7.29	0.00	1.73
B. Recovery Period	PLA				CA + C			
	R1	R2	*p*-value	Cohen’s d	R1	R2	*p*-value	Cohen’s d
Heart Rate (bpm)	102.56 ± 10.79	87.6 ± 11.30	0.00	1.35	111.56 ± 11.37	94.4 ± 9.77	0.00	1.35
lnRMSSD (ms)	1.42 ± 0.49	2.54 ± 0.66	0.00	1.90	1.51 ± 0.84	2.50 ± 0.68	0.03	1.29
lnSDNN (ms)	2.90 ± 0.31	3.57 ± 0.49	0.01	1.62	2.89 ± 0.45	3.71 ± 0.58	0.01	1.59
lnHF (ms^2^)	1.59 ± 0.62	3.92 ± 1.30	0.00	2.30	1.50 ± 1.50	4.23 ± 1.22	0.00	2.01
HFnu	12.33 ± 7.30	13.61 ± 11.11	0.78	0.14	19.37 ± 20.31	18.00 ± 13.06	0.71	0.08
lnLF (ms^2^)	3.69 ± 0.97	6.04 ± 1.26	0.00	2.09	3.19 ± 1.12	5.71 ± 1.50	0.00	1.90
LFnu	87.62 ± 7.27	86.37 ± 11.14	0.78	0.13	80.45 ± 20.50	81.95 ± 13.11	0.69	0.09
LF/HF	9.69 ± 4.83	12.18 ± 12.00	0.47	0.27	7. 63 ± 4.22	7.18 ± 5.15	0.82	0.09
E (nmol/L)	31.50 ± 19.09	4.22 ± 2.91	0.00	2.00	38.24 ± 26.44	5.50 ± 2.34	0.00	1.74
NE (nmol/L)	158.20 ± 82.26	30.11 ± 14.08	0.00	2.17	179.14 ± 61.37	35.20 ± 8.51	0.00	3.2

Chronotropic markers of ANS activity is represented as HR; Markers of Heart Rate Variability (HRV) are the log transformed mean square of successive N-N intervals (lnRMSSD), standard deviation of the N-N intervals (lnSDNN), the High Frequency (lnHF), Low Frequency (lnLF) and the ratio (LF/HF); Plasma Catecholamines are represented as Epinephrine (E) and Norepinephrine (NE). All data are presented as Means ± SD

≤ 0.05 significant time effects

## Discussion

The purpose of this study was to examine the resting ANS response to the ingestion (I1 - I2) of CA + C as well as its influence on the ANS response to an exhaustive exercise protocol (R1 - R2). During the ingestion period HR, lnLF, LFnu, E and NE significantly increased in the CA + C trial, indicating an enhanced sympathetic response to the supplementation. Interestingly, a significant decrease in HFnu was observed while no changes in lnHF or lnRMSSD occurred despite a significant increase in lnSDNN. All cardiac autonomic markers demonstrated a time-dependent shift towards baseline following the exercise protocol except for LFnu, HFnu, and LF/HF. Further points of consideration are provided below.

### Ingestion period

Resting HR is a primary marker in cardiac autonomic activity and is affected through several intrinsic and extrinsic factors; however, the influence of the combination of CA + C on resting HR is relatively unknown. The limited amount of information available pertains to the isolated components CA and C, with only one known study to have examined the combination of both [2]. For instance, Min et al., [10] examined only p-synephrine, the active component of CA, and found no changes in resting HR at one-hour, three-hours, six-hours, or eight-hours following consumption. Furthermore, recent studies have shown little no changes in resting HR with caffeine consumption alone in habitual caffeine consumers [27, 28]. When combining a 100 mg of CA, and 100 mg of C, Ratamess et al. [2] observed no significant changes in resting HR following a three-week washout period from caffeine when compared to controls. In contrast, the findings of the current study demonstrated a time-dependent increase in HR following the consumption of the CA + C complex. No time-dependent changes were observed during the ingestion phase for the PLA trial. This discrepancy may be due to differences in the experimental design. For instance, the participants in the Ratamess et al. [2] study underwent a three day exposure to the CA + C complex prior to testing, while in the current study participants were not given the CA + C complex prior to the experimental trials, which may account for the difference in the response.

Overall, the evaluation of CA or CA + C on markers of ANS activity is currently understudied, but information regarding other stimulants (e.g. caffeine) is replete [3–5, 29, 30]. For instance, Rauh et al. [31] found that the consumption of either 100 mg or 200 mg of caffeine in habitual users failed to alter any examined marker of HRV. Zimmerman et al. [28] examined caffeine consumption in both habitual and non-habitual consumers and found no effect on SNS related activity (LF and LF/HF), but enhanced PNS activity (RMSSD and HF) in habitual users was observed. Conversely, Yoshinaga et al., observed significant increases in the power spectral density in both LF and HF following the ingestion of 4 mg•kg of bodyweight [32]. The findings of the current study provide equivocal results to the literature, where the consumption of CA + C increased HR, lnLF, LFnu, E, and NE; indicating an increase in SNS activity while showing no changes in PNS markers (lnRMSSD and lnHF) during the ingestion period. Interestingly, a nonsignificant rise of lnLF and reduction of lnHF was observed following the PLA trial. However, when evaluating LFnu and HFnu a similar yet significant changed was observed, demonstrating a relative change in the ratios rather than the absolute values of the power spectral density. This may in part be due to the anticipation of the upcoming exhaustive protocol and pre-performance anxiety, resulting in minor shifts of ANS activity. Future studies should evaluate and account for pre trial emotional stress.

When evaluating plasma markers of SNS activity, it has been proposed that circulating sympathetic biomarkers E and NE increase following consumption of caffeine [33, 34]; however, a recent study demonstrated caffeine to have little to no influence over resting values [35]. The observed changes in E and NE within the current study support the previous notion that caffeine influences resting plasma levels and reflect the changes observed in SNS related HRV markers following the CA + C ingestion. The lack of change in the lnRMSSD and lnHF in the presence of increased SNS activity acts against the traditional interplay between PNS and SNS balance. Generally, with increases in SNS activity a withdrawal of vagal tone occurs. However, this was not observed and could be the result of a decreased sensitivity to caffeine or the rested state of the participant, which resulted in the attenuation of vagal activity.

Regardless of the mechanisms involved, the overall cardiac autonomic response observed during the ingestion phase is suggestive of a “priming” of the SNS response to the CA + C supplementation. Traditionally, it is believed that ANS activity is balanced between the PNS and SNS branches, exhibiting an inverse relationship [12]. However, the PNS/SNS interplay appears to be more complex, and likely exhibits their influences upon each other on a spectrum, rather than direct counter balance. The findings of this study are indicative of this type of relationship between the SNS and PNS branches, specifically the lack of change in resting PNS activity when compared to the time-based increases in SNS drive observed in the CA + C trial. Specifically, there was an increase in lnSDNN and lnLF without the presence of altered vagal activity. This is important because these markers are believed to have influences stemming from both the SNS and PNS [36]. Therefore, with no discernable changes within markers of vagal activity, it can be inferred that the increases of lnLF and lnSDNN are the result of changes seen in SNS activity. This response adds to the understanding of the level of complexity within ANS control and should be further investigated to determine thresholds between the various markers.

### Recovery period

Following the exhaustive protocols, HR was significantly elevated in both trials and recovered in a similar time-dependent fashion (Table 2A and B). This is consistent with the findings of Haller et al. [37] who did not observe any differences in post-exercise recovery of HR when comparing a commercially available supplement (21 mg of synephrine, 303.8 mg of caffeine, and various additives) to a placebo during the 30-min to 12-h following a moderate exercise bout [37]. Additionally, markers of HRV following the exhaustive protocol demonstrated nearly identical physiological responses, with a decrease in activity post exercise and a gradual increase toward baseline values, which is a commonly observed post exercise response [11, 38]. A similar yet inverse response was observed in circulating plasma E and NE, with substantial increases post exercise and a return to baseline values within 45-min post (R2). It is important to note that though increased SNS activity was observed following the ingestion of CA + C, the effect was lost following the exhaustive protocol. Therefore, the findings are suggestive that the influence of CA + C occurs only in a resting environment and does not influence the recovery of the system.

Though it was outside the scope of the current study to establish mechanisms involved with CA + C consumption, we will postulate on potential factors involved in the observed findings. The ingestion phase (I1 - I2) at rest was the only period in which CA + C appeared to have an effect on SNS activity. As previously mentioned, recent studies have demonstrated that C provides little cardiovascular stimulation and more so acts to improve PNS activity rather than inhibit in habitual users [28]. However, it should not be overlooked that the research is conflicting in habitual consumers and that C has been shown to alter SNS activity through increased sensitivity to circulating E and NE [29]. Within the current study, plasma E and NE significantly increased following CA + C ingestion in our habitual C population (209 ± 95.5 mg/day). There was no withdrawal of PNS activity despite a significant increase in resting HR, which could be explained by the combined effects of circulating E and NE as well as improved sensitivity related to C. The acting ingredient of CA, p-synephrine, works primarily on the ß-3 receptors on adipose tissue and therefore is unlikely to have any direct impact on autonomic function [7]. Indirectly, the increased activity of lipolysis, and thermogenesis caused by p-synephrine could have elevated SNS activity, which was demonstrated by Reimann et al. [39] who found that acute hyperlipidemia increased both HR and sympathetic drive. Though we did not measure changes in plasma lipids we can postulate that the known action of p-synephrine could have elevated plasma levels and consequently influenced sympathetic activity.

## Conclusion

In summary, the CA + C complex (100 mg each) used in this study was enough to elicit what can best be described as a priming of the SNS response after consumption. As such, the physiological aspects involved with CA + C supplementation appear to have the greatest influence in a resting environment. Beyond modest increases in SNS activity at rest, little benefit was observed during the exhaustive protocol recovery period, which was the primary purpose of the investigation. Therefore, the authors conclude that in healthy male participants who regularly consume caffeine, CA + C does not improve recovery of cardiac autonomic function following an exhaustive exercise protocol. The observed priming of the SNS activity with no alterations of PNS activity provided new insight into the complex relationship of the ANS and warrants further investigation.

## Abbreviations

ANS: Autonomic nervous system
Body Fat: BF%
C: Caffeine
CA + C: Caffeine and *Citrus aurantium* Complex
CA: *Citrus aurantium*
CNS: Central nervous system
E: Epinephrine
HCT: Hematocrit
HF: High frequency
HFnu: High frequency normalized
HHQ: Health history questionnaire
HR: Heart rate
HRV: Heart rate variability
LF: Low frequency
LF/HF: Low frequency/ High frequency ratio
LFnu: Low frequency normalized
NE: Norepinephrine
PAR-Q: Physical activity readiness questionnaire
PLA: Placebo
PNS: Parasympathetic nervous system
RMSSD: Root mean square of successive R-R intervals
SDNN: Standard deviation of N-N intervals
SNS: Sympathetic nervous system
